# Few-shot object detection for pest insects via features aggregation and contrastive learning

**DOI:** 10.3389/fpls.2025.1522510

**Published:** 2025-06-19

**Authors:** Shuqian He, Biao Jin, Xuechao Sun, Wenjuan Jiang, Jiaxing Gu, Fenglin Gu

**Affiliations:** ^1^ School of Information Science and Technology, Hainan Normal University, Haikou, Hainan, China; ^2^ Hainan Provincial Engineering Research Center for Artificial Intelligence and Equipment for Monitoring Tropical Biodiversity and Ecological Environment, Hainan Normal University, Haikou, Hainan, China; ^3^ College of Computer Science and Technology, Zhejiang University, Hangzhou, Zhejiang, China; ^4^ Spice and Beverage Research Institute, Chinese Academy of Tropical Agricultural Sciences, Wanning, Hainan, China

**Keywords:** feature aggregation, contrastive learning, few-shot learning, object detection, pest control

## Abstract

Accurate detection of pest insects is critical for agricultural pest management and crop yield protection, yet traditional detection methods struggle due to the vast diversity of pest species, significant individual differences, and limited labeled data. These challenges are compounded by the typically small size of pest targets and complex environmental conditions. To address these limitations, this study proposes a novel few-shot object detection (FSOD) method leveraging feature aggregation and supervised contrastive learning (SCL) within the Faster R-CNN framework. Our methodology involves multi-scale feature extraction using a Feature Pyramid Network (FPN), enabling the capture of rich semantic information across various scales. A Feature Aggregation Module (FAM) with an attention mechanism is designed to effectively fuse contextual features from support and query images, enhancing representation capabilities for multi-scale and few-sample pest targets. Additionally, supervised contrastive learning is employed to strengthen intra-class similarity and inter-class dissimilarity, thereby improving discriminative power. To manage class imbalance and enhance the focus on challenging samples, focal loss and class weights are integrated into the model’s comprehensive loss function. Experimental validation on the PestDet20 dataset, consisting of diverse tropical pest insects, demonstrates that the proposed method significantly outperforms existing approaches, including YOLO, TFA, VFA, and FSCE. Specifically, our model achieves superior mean Average Precision (mAP) results across different few-shot scenarios (3-shot, 5-shot, and 10-shot), demonstrating robustness and stability. Ablation studies confirm that each component of our method substantially contributes to performance improvement. This research provides a practical and efficient solution for pest detection under challenging conditions, reducing dependency on large annotated datasets and improving detection accuracy for minority pest classes. While computational complexity remains higher than real-time frameworks like YOLO, the significant gains in detection accuracy justify the trade-off for critical pest management applications.

## 1 Introduction

Accurate detection of pest insects is crucial for effective pest management and agricultural productivity. Pest infestations can cause significant crop losses, threatening food security and economic stability worldwide. Traditional detection methods typically rely on manual inspection, which is time-consuming, labor-intensive, and prone to human error. With advancements in computer vision and deep learning, automated pest detection systems have gained attention for their potential to offer rapid and accurate identification of pest species in real-world agricultural environments (Rai and Sun, 2024). Developing robust pest detection models, however, remains challenging for several reasons. First, pest insects exhibit high intra-class variability (e.g., different developmental stages such as eggs, larvae, pupae, and adults) and low inter-class variability (similar appearances across species). This contrast often complicates accurate feature extraction and classification (Butera et al., 2021; Popescu et al., 2023). Second, constructing large-scale annotated datasets is difficult because gathering and labeling images for numerous pest species is resource-intensive and requires domain expertise. (Li, Y et al., 2021; Yang et al., 2022). In pest object detection, there are a huge number of pest types, and it is extremely costly or even impossible to directly detect all species. Collecting large-scale pest datasets is also highly challenging. In practice, pest management predominantly targets crops, with timely response to primary pests being essential (Ali et al., 2024). Rapidly collecting a small number of samples for these major pests can be more practical and cost-effective. Therefore, few-shot object detection (FSOD) has significant research value in pest management, as it enables effective detection of critical pests with minimal annotated data.

Few-shot learning (FSL) has emerged as a promising solution to address the problem of limited annotated data (Li, Y et al., 2021; Li X. et al., 2023). FSL aims to recognize new classes using only a few labeled examples by leveraging prior knowledge learned from other tasks or classes. In the context of pest detection, FSL can enable models to identify novel pest species with minimal labeled samples, which is highly valuable for practical agricultural applications. Despite the progress in FSL for image classification tasks, applying FSL to object detection, especially for small and densely packed pest insects, remains a significant challenge (Huang et al., 2023; Pöhler et al., 2023). Traditional object detection models like Faster R-CNN (Ren et al., 2016) struggle with small objects due to insufficient feature representation and the dominance of background information (Teng et al., 2022). Moreover, the high similarity between different pest species further complicates accurate detection and classification.

To overcome these challenges, we propose a novel FSOD framework specifically designed for pest insects, integrating feature aggregation and contrastive learning techniques. Our approach builds upon the Faster R-CNN architecture and introduces a Feature Aggregation Module (FAM) that leverages multi-scale features from both support and query images. By employing an attention mechanism, the model effectively fuses rich contextual information from the support set to enhance the representation of multi pest objects in the query images. Additionally, we incorporate SCL to improve the discriminative ability of the model. Contrastive learning has shown effectiveness in enhancing feature representations by pulling together samples of the same class and pushing apart samples of different classes (Sun et al., 2021). By integrating contrastive learning into the detection framework, we aim to increase intra-class compactness and inter-class variance, which is crucial for distinguishing between visually similar pest species.

Furthermore, we address the issue of class imbalance inherent in pest detection datasets by introducing a balancing mechanism in the loss function. We adopt the focal loss to focus the training on hard examples and underrepresented classes, thereby improving the model’s robustness and accuracy (Li, Y et al., 2021; Wen et al., 2022).

### 1.1 Key contributions include

#### 1.1.1 Feature aggregation module

We design a novel Feature Aggregation Module (FAM) that enhances the representation of multiple pest objects by aggregating multi-scale features from support and query images using an attention mechanism.

#### 1.1.2 Supervised contrastive learning

We integrate SCL into the object detection framework to improve feature discrimination, promoting intra-class similarity and inter-class dissimilarity among pest species.

#### 1.1.3 Balancing mechanism

We introduce a balancing mechanism in the loss function using focal loss to mitigate the impact of class imbalance in pest detection datasets.

#### 1.1.4 Comprehensive evaluation

We conduct extensive experiments on benchmark pest detection datasets to validate the effectiveness of our proposed method, demonstrating significant improvements over baseline models.

The proposed method provides a practical solution for agricultural pest management by enabling accurate detection of critical pests with minimal annotated data (Ragu and Teo, 2023). Its ability to handle few-shot scenarios ensures timely responses to pest outbreaks, reducing reliance on pesticides and promoting sustainable practices.

The remainder of this paper is organized as follows: Section 2 reviews related work on pest detection, few-shot learning, and contrastive learning. Section 3 details our proposed methodology, including the Feature Aggregation Module (FAM), the SCL approach, and the multi-task loss function. Section 4 presents experimental setups and results, and Section 5 concludes with future directions for research.

## 2 Related work

### 2.1 Pest detection in agriculture

The application of deep learning techniques in agriculture, particularly for pest detection, has gained momentum in recent years (Popescu et al., 2023; Mahmood et al., 2023). Traditional pest detection methods often rely on manual scouting, which is inefficient and prone to human error (Butera et al., 2021). Deep learning-based approaches offer automated, accurate, and real-time detection capabilities, vital for integrated pest management systems.

Several studies have focused on object detection models tailored for pest insects. For instance, Pang et al. (2022) proposed an improved YOLOv4 algorithm for real-time pest detection in orchards, reportedly achieving high detection accuracy (mAP above 80%) with efficient processing speeds. Similarly, Wen et al. (2022) introduced Pest-YOLO to detect dense, tiny pests, attaining about 92% detection accuracy on large-scale datasets.

Despite these successes, both methods relied on substantial annotated data, which is often infeasible given the vast diversity of pest species and the complexity of field conditions (Liu et al., 2022). Moreover, many pests are small or densely clustered, challenging conventional detectors that struggle with small-object detection (Teng et al., 2022; Yang et al., 2024).

### 2.2 Few-shot learning in agriculture

Few-shot learning (FSL) has emerged as a solution to data scarcity. In agriculture, FSL has been applied to tasks like plant disease recognition (Li, Y et al., 2021; Yang et al., 2022; Argüeso et al., 2020; Chen et al., 2021) and pest detection (Li X. et al., 2023; Rezaei et al., 2024; Li Y. et al., 2023). Li and Chao (Li and Chao, 2021) proposed a semi-supervised few-shot learning approach for plant disease recognition, leveraging unlabeled data to improve classification when labeled samples are limited. Yang et al. (2022), Cao et al. (2023), Liang et al. (2021), Lin et al. (2024) and Lin et al. (2022a) highlighted the role of FSL in smart agriculture, noting its effectiveness for rapid adaptation to new conditions or pest species. In pest detection, few-shot learning enables models to generalize to new pests with only a handful of labeled samples, a crucial capability given the difficulty of obtaining comprehensive data for every pest species. Li X. et al. (2023) introduced a few-shot crop pest detection method using object pyramids, reporting a notable increase in mAP under low-data conditions. Rezaei et al. (2024), Egusquiza et al. (2022) and Zhou et al. (2023) demonstrated that even modest improvements in few-shot scenarios significantly impacted real-world applications, reinforcing the practicality of FSL in pest management (Gao et al., 2024). Nonetheless, effectively transferring FSL methods from classification to object detection remains challenging (Wang C. et al., 2023; Wang et al., 2021), especially under severe data constraints and small-object settings.

### 2.3 Contrastive learning and feature representation

Contrastive learning has gained attention for learning discriminative feature representations by contrasting positive and negative sample pairs (Sun et al., 2021). In FSOD, contrastive learning helps models differentiate classes with limited samples by enlarging inter-class separation within the feature space. Sun et al. (2021). proposed FSCE, which encodes proposals using contrastive learning to enhance detection performance in few-shot settings, reportedly improving mAP on benchmark datasets by up to 3–5 percentage points. In agricultural applications, contrastive learning has also been employed to improve classification. Song et al. (2023) and Zhong et al. (2020) used an attention-based generative adversarial network with few-shot learning to boost feature representation for maize disease detection, achieving higher accuracy scores compared to baseline CNN models. These results suggest that contrastive learning can likewise benefit the detection of various agricultural pests, particularly when data are limited or imbalanced.

### 2.4 Feature aggregation techniques

Feature aggregation combines features from different layers or sources to improve detection performance. For small-object detection, multi-scale feature fusion can be critical (Kong et al., 2024; Lin et al., 2022b). Teng et al. (2022) developed MSR-RCNN, integrating multi-scale super-resolution enhancements, increasing detection accuracy for small pest objects by around 4% in mAP. Han J. et al. (2023) presented a FSOD method using variational feature aggregation, demonstrating substantial improvements under limited-data conditions.

### 2.5 Addressing class imbalance

Class imbalance is pervasive in pest detection, where certain dominant pest species overshadow minority ones (Wen et al., 2022; Liu et al., 2022). Focal loss has proven effective in re-weighting hard examples and mitigating bias toward majority classes (Li, Y et al., 2021; Wen et al., 2022). Anwar and Masood (Anwar and Masood, 2023) also emphasized the importance of addressing imbalance, demonstrating a 5-8% improvement in detection accuracy by incorporating focal loss and augmenting minority classes.

### 2.6 Advances in few-shot object detection

Recent surveys by Huang et al. (2023) and Pöhler et al. (2023) extensively review FSOD methods, including meta-learning, transfer learning, and metric learning techniques. The Segment Anything Model (SAM) (Zhang et al., 2023) represents a significant advancement in vision models, generalizing to new tasks with minimal data. While SAM primarily targets segmentation, it could be adapted for object detection under few-shot scenarios. Further, Han et al. (2023) extended SAM to open-vocabulary learning, enabling zero-shot generalization to unseen classes. These advancements suggest promising directions for applying cutting-edge few-shot methods to pest detection tasks.

Despite these advancements, several challenges persist in pest detection. First, multi-object detection remains problematic, as many models fail to handle multiple, densely packed pest insects due to insufficient feature representation (Teng et al., 2022; Yang et al., 2024). Second, labeled data scarcity restricts models from generalizing to novel pests, especially when each species demands expert-labeled samples (Li, Y et al., 2021; Yang et al., 2022; Liu et al., 2022). Third, visual similarity among pests complicates accurate feature discrimination (Butera et al., 2021; Popescu et al., 2023). Finally, class imbalance skews detection results, disadvantaging minority species (Wen et al., 2022; Liu et al., 2022; Wang X. et al., 2023). Our proposed method addresses these issues by incorporating feature aggregation to improve multi-object representation, SCL to enhance feature discrimination for visually similar pests, and a balancing mechanism to correct dataset imbalance.

In doing so, we aim to advance the state of pest detection by boosting accuracy for small, minority-class targets, reinforcing the practicality of few-shot techniques in agricultural domains.

### 2.7 Comparison of existing pest recognition methods

As shown in **Table 1**, the comparison table summarizes various pest recognition methods, highlighting the differences in tasks, architectures, and small-shot learning capabilities. Previous research on pest identification and detection largely relied on CNN-based architectures, including YOLO and Faster R-CNN, which offered effective solutions for recognizing and localizing pests but struggled with challenges like detecting tiny pests, distinguishing visually similar species, and addressing class imbalance. Although recent works introduced improvements, such as multi-scale feature fusion, super-resolution sampling, and focal-loss-based imbalance handling, they generally addressed these issues separately rather than in a unified framework. Few-shot methods, while beneficial for scenarios with limited training data, were often limited to classification tasks without explicit handling of small pests or class imbalance. In contrast, the method proposed in this paper innovatively integrates multi-scale feature aggregation, supervised contrastive learning, and focal loss within a unified Faster R-CNN framework. Feature aggregation significantly improves multi-object detection by fusing multi-scale features, while supervised contrastive learning enhances discriminative capabilities by effectively differentiating similar pest species even from minimal examples. Additionally, focal loss addresses class imbalance by prioritizing minority-class and challenging samples during training. Consequently, this comprehensive approach robustly tackles key limitations of existing methods, achieving superior detection accuracy and better generalization to novel and rare pest species, demonstrating significant practical value for real-world agricultural applications under limited labeled data conditions.

**Table 1:** Comparison of existing pest recognition methods.

Function	Architecture	Contrastive learning	Multi-target and multi-scale	Class imbalance handling	Representative papers
Recognition (Classification)	Deep CNNs	No	multi-scale	No	Popescu et al. (2023)
	Deep CNNs with ensemble-based mode	No	No	No	Anwar and Masood (2023)
	CNN+Transformer	No	No	No	An et al. (2023)
	Transformer+super resolution sampling technique	No	No	No	Bai et al. (2023)
Recognition (Classification) Few-Shot	Transformers	No	No	No	uthalapati and Tunga (2021)
	a multi-layer feature fusion (FMLF) method	No	Yes	No	Gomes et al. (2023)
Object Detection (Classification/position)	Deep CNNs	No	multi-scale	No	Butera et al. (2021)
	YOLO	No	Multi-target and multi-scale	No	Pang et al. (2022)
	Faster R-CNN	No	multi-scale	No	Wang C. et al. (2023)
	Pest-YOLO	No	No	Yes	Wen et al. (2022)
	multi-scale super-resolution RCNN	No	multi-scale	No	Teng et al. (2022)
	SRNet-YOLO	No	multi-scale	No	Yang et al. (2024)
Object Detection (Classification/position) Few-Shot	Faster R-CNN	No	multi-scale	No	Li X. et al. (2023)
	Faster R-CNN	No	multi-scale	No	Yang et al. (2023)
	Faster R-CNN	No	multi-scale	No	Wang X. et al. (2023)
	Faster R-CNN	Yes	Multi-target and multi-scale	Yes	Proposed Method

## 3 Proposed methodology

Our research presents an improved model based on the Faster R-CNN framework, aiming to enhance the feature representation capability of small-sample targets and improve object detection performance. Traditional Faster R-CNN frameworks face performance bottlenecks when handling small samples and multi object detection, primarily due to limitations in feature extraction layers and insufficient representation of small object features. To address these issues, we introduce multi-scale feature extraction for the support set and query set, expanding the capacity of feature extraction.

After feature extraction, the model inputs the features of the support set and query set into the Feature Aggregation Module (FAM). This module employs an attention mechanism for relational modeling, calculating the correlation between the support set and query set to construct aggregated features for multi-scale and multi objects. This feature aggregation method effectively utilizes the rich feature information from the support set, enhancing the feature representation capability of the query set, especially for detecting small-sample targets.

To further improve the model’s discriminative ability, we incorporate SCL. By performing contrastive learning mapping and normalization on features, we enhance intra-class similarity and inter-class dissimilarity, promoting the clustering of similar samples and the separation of dissimilar samples in the feature space, thereby alleviating misclassification issues. However, SCL may suffer from sample imbalance problems, where insufficient samples of minority classes may cause the model to bias toward majority classes. To resolve this, we introduce an imbalance correction mechanism, adopting Focal Loss to optimize the loss function, assigning higher weights to hard-to-classify samples, and balancing the influence of each class.

Finally, we adopt a multi-task learning approach to jointly optimize four tasks: localization, classification, feature aggregation, and SCL. By integrating these components into the model, we achieve efficient detection of multi-sample targets, enhancing the model’s feature representation capability and classification accuracy.

### 3.1 Framework overview

**Figure 1:**
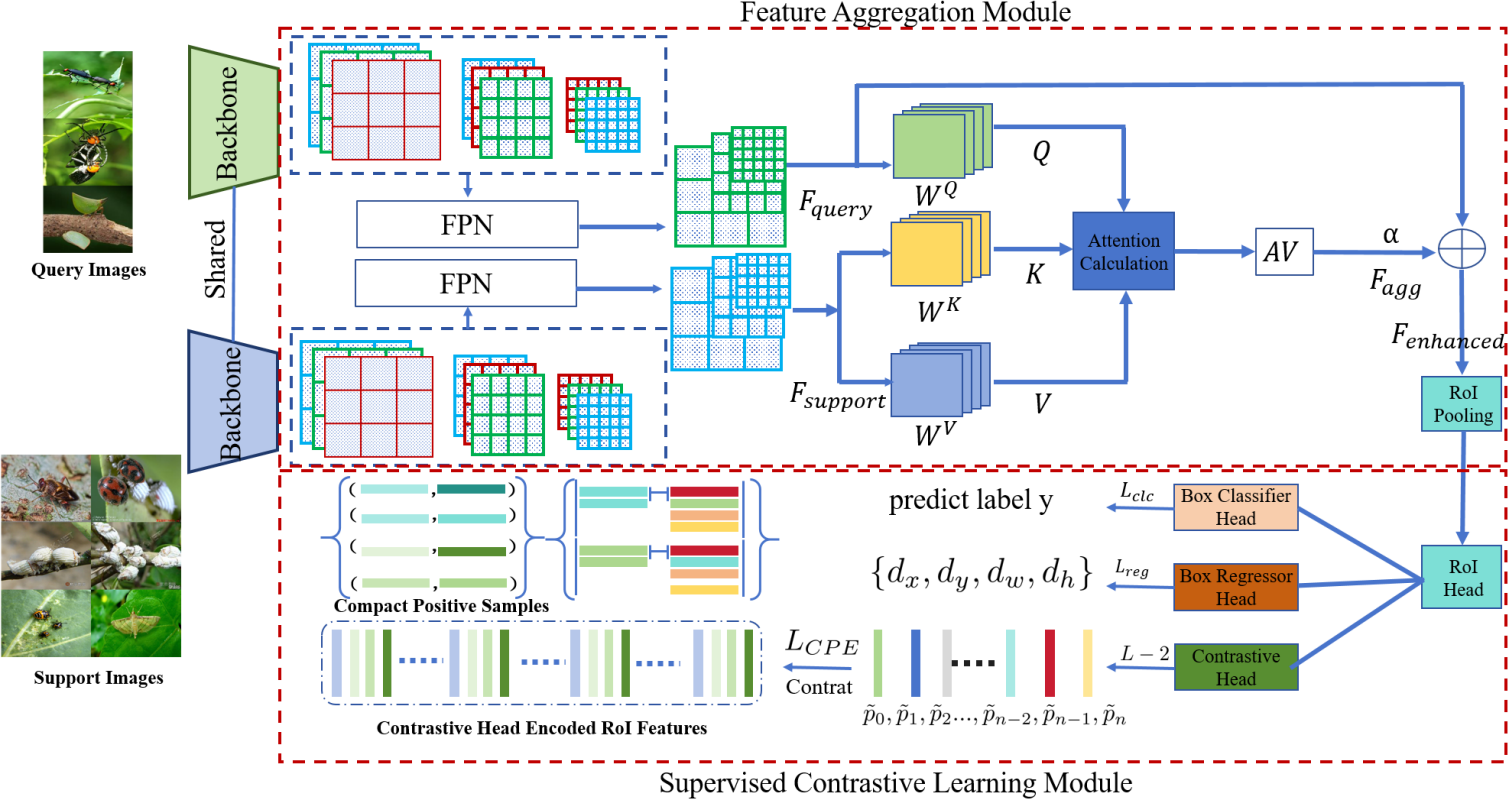
Overall model architecture.

As shown in **Figure 1**, our proposed model is built on the Faster R-CNN framework and is enhanced to effectively represent the features of multi-sample objects. The model architecture comprises several key components: first, multi-scale feature extraction, which integrates a Feature Pyramid Network (FPN) into the backbone network to capture rich information across various scales. Second, the Feature Aggregation Module (FAM), an attention-based component, aggregates features from both the support set and query set, enhancing the representation of multi-scale objects. Third, the SCL module improves the discriminative ability of the feature space by maximizing intra-class similarity and inter-class differences. Fourth, an imbalance correction mechanism incorporates focal loss into the loss function to address sample imbalance, ensuring the model focuses more on minority classes and challenging examples. Finally, the multi-task learning optimization jointly optimizes localization, classification, feature aggregation, and contrastive learning tasks through a comprehensive loss function. This integration enables the model to exploit contextual and class-specific information from the support set, significantly improving detection performance for both multi-object and few-shot objects. While Faster R-CNN is known to struggle with small-object detection due to insufficient feature representation, it was chosen for its robust two-stage detection process, which ensures precise localization and classification. The integration of FAM and SCL addresses its limitations by enhancing feature representation and improving discrimination for small objects. Comparative results show that the proposed enhancements improve mAP for small objects compared to the unmodified Faster R-CNN.

### 3.2 Feature aggregation module

The core objective of the Feature Aggregation Module (FAM) is to utilize the rich feature information from the support set to enhance the feature representation capability of the query set, especially for detecting multi and multi-scale objects. Traditional feature extraction methods have limited ability to represent multi object features, whereas the support set provides additional context and class information to compensate for this deficiency.

#### 3.2.1 Multi-scale feature extraction

We integrate a Feature Pyramid Network (FPN) into the backbone network to extract features from different scales. Specifically, we obtain feature maps from multiple levels (C2, C3, C4, C5) of the backbone network (e.g., ResNet) and generate multi-scale feature maps $\left\{P_{2},P_{3},P_{4},P_{5},P_{6}\right\}$ through 1×1 and 3×3 convolution operations. This multi-scale feature extraction ensures the model’s sensitivity to targets of various sizes.

For each Region of Interest (RoI) in the support set and query set, we perform RoI Align operations on these multi-scale feature maps to obtain fixed-size feature representations (e.g., 7×7). These feature representations preserve spatial information and contextual relationships, providing rich features for subsequent feature aggregation.

#### 3.2.2 Structure of the feature aggregation module

The Feature Aggregation Module (FAM) consists of the following components: Feature Mapping, maps the features of the support set and query set into query (Q), key (K), and value (V) spaces, as shown in Equation 1. Attention Mechanism, calculates the similarity between queries and keys to obtain the attention weight matrix. Feature Fusion, uses attention weights to perform weighted summation of values, achieving feature aggregation.

Implementation Details:

##### 3.2.2.1 Mapping features to query, key, and value spaces

First, we map the features of the support set and query set into low-dimensional spaces through linear transformations as shown in Equation 1:


(1) $$Q=F_{query}W^{Q},\;K=F_{support}W^{K},\;V=F_{support}W^{V}$$

Where $F_{query}$ and $F_{support}$ are the feature representations of the query set and support set, respectively, and $W^{Q}$ , $W^{K}$ and $W^{V}$ are learnable parameter matrices.

##### 3.2.2.2 Calculating attention weights

Using the dot product between queries and keys, we calculate the similarity scores and normalize them through the softmax function as shown in Equation 2:


(2) $$\text{A}=\text{softmax,}\;(\frac{{\text{QK}}^{⊤}}{\sqrt{{\text{d}}_{\text{k}}}})$$

where ${\text{d}}_{\text{k}}$ is the dimension of the key vectors, used to scale the dot product to prevent excessively large values.

##### 3.2.2.3 Feature aggregation

Using the attention weight matrix A to perform weighted summation of the values V, the aggregated feature representation is formulated as Equation 3.


(3) $$F_{agg}=AV$$

Then, we fuse the aggregated features with the original query set features to obtain the enhanced feature representation, defined in Equation 4.


(4) $$F_{enhanced}=F_{query}+\text{α}F_{agg}$$

Where α is a learnable scaling factor that controls the influence of the aggregated features on the original features.

#### 3.2.3 Aggregated features for multi-scale and multi objects

Through the feature aggregation process described above, the feature representation of the query set is enhanced in several ways. Multi-scale information fusion leverages features from the support set’s multi-scale feature maps, providing rich scale information that aids in detecting targets of various sizes. Multi object feature enhancement is achieved by supplementing high-level feature maps with low-level features from the support set, which preserves details that are often lost for multi objects. Additionally, the support set’s features provide valuable contextual information, helping the model understand the relationship between the target and its background. The module offers several advantages: it improves feature representation by effectively utilizing the rich information from the support set, enhances flexibility and scalability through an attention-based relational modeling approach that adaptively adjusts the influence of the support set on the query set, and allows for easy integration into existing object detection frameworks with minimal computational overhead.

### 3.3 Supervised contrastive learning module

In object detection tasks, the model’s discriminative ability is crucial for detection accuracy. However, due to the dispersion of intra-class features and the overlap of inter-class features, the model may experience misclassification issues. To address this problem, we introduce SCL, aiming to optimize the feature space so that features of the same class are closer together, while features of different classes are farther apart.

#### 3.3.1 Contrastive learning feature mapping and normalization

We apply a projection head Head(·) to the enhanced features $F_{enhanced}$ output from the Feature Aggregation Module (FAM) to map them into the contrastive learning feature space, as expressed by Equation 5.


(5) $${\text{z}}_{\text{i}}=\text{Normalize}(\text{Head}({\text{F}}_{\text{enhanced},\text{i}}))$$

Where Normalize (·) denotes ${\text{L}}_{2}$ normalization to ensure the feature vectors lie on a unit hypersphere.

#### 3.3.2 Contrastive learning feature mapping and normalization

In SCL, label information is used to construct positive and negative sample pairs. Positive samples consist of a query sample *i* and support samples p∈P(i) that belong to the same class. Negative samples consist of the query sample *i* and support samples a∈A(i) from different classes. Here, P(i) represents the set of samples in the same class as sample *i*, while A(i) represents the set of all samples except sample *i*.

#### 3.3.3 Supervised contrastive loss function

We adopt the supervised contrastive loss function to optimize the feature representation, which is formulated as Equation 6.


(6) $${\text{L}}_{\text{SCL}}=\sum_{\text{i}\in \text{I}}\frac{1}{\left|\text{P}(\text{i})\right|}\sum_{\text{p}\in \text{P}(\text{i})}-\log(\frac{\exp({\text{z}}_{\text{i}}\cdot {\text{z}}_{\text{p}}/\text{τ})}{\sum_{\text{a}\in \text{A}(\text{i})}\exp({\text{z}}_{\text{i}}\cdot {\text{z}}_{\text{a}}/\text{τ})})$$

Where τ is the temperature parameter controlling the smoothness of the distribution.

By minimizing the supervised contrastive loss, the model is guided to achieve intra-class compactness, where features of the same class are closer together, enhancing similarity within each class. It also promotes inter-class separation, pushing features of different classes farther apart and increasing dissimilarity between classes. This optimization helps the model classify more accurately and reduces misclassification.

#### 3.3.4 Correction for sample imbalance

SCL may be affected by sample imbalance, where minority classes have insufficient samples, causing the model to bias toward majority classes. To address this, we introduce an imbalance correction mechanism.

Specifically, we incorporate class weights ${\text{w}}_{{\text{y}}_{\text{i}}}$ into the supervised contrastive loss, adjusting according to the number of samples in each class, as defined in Equation 7.


(7) $$w_{y_{i}}=\frac{1}{N_{y_{i}}}$$

where ${\text{N}}_{{\text{y}}_{\text{i}}}$ is the number of samples in class ${\text{y}}_{\text{i}}$ . The loss function Equation 6 becomes Equation 8.


(8) $$L_{\text{SCL}}=\sum_{i\in I}\frac{w_{y_{i}}}{\left|P(i)\right|}\sum_{p\in P(i)}-\log(\frac{\exp(z_{i}\cdot z_{p}/\text{τ})}{\sum_{a\in A(i)}\exp(z_{i}\cdot z_{a}/\text{τ})})$$

This adjustment prioritizes minority class samples in the loss function, prompting the model to focus more on learning these classes.

The introduction of SCL will enhance the discriminability of the feature space, reduce misclassification, and thus improve classification accuracy; at the same time, through the imbalance correction mechanism, the model can learn the minority classes more fully and adapt to imbalanced data; finally, better feature representation helps the model perform better on unknown data and enhances generalization capabilities.

### 3.4 Overall loss function design

#### 3.4.1 Construction of the multi-task loss function

To jointly optimize the model’s components, we design a comprehensive multi-task loss function that includes localization loss, classification loss, feature aggregation loss, and supervised contrastive loss, which is defined in Equation 9.


(9) $$L_{\text{total}}=L_{\text{cls}}+L_{\text{reg}}+{\text{λ}}_{1}L_{\text{agg}}+{\text{λ}}_{2}L_{\text{SCL}}$$

Among them, $L_{\text{cls}}$ is the classification loss, which measures the model’s prediction accuracy of the target class, $L_{\text{reg}}$ is the regression loss, which measures the model’s positioning accuracy of the target bounding box, $\;L_{\text{agg}}$ is the loss of the Feature Aggregation Module (FAM), which may include the regularization term of the attention mechanism, $L_{\text{SCL}}$ is the supervised contrast loss, which enhances the discriminability of feature representation, ${\text{λ}}_{1}{\text{ and λ}}_{2}$ are trade-off coefficients that adjust the impact of each loss term.

#### 3.4.2 Design of the classification loss

We employ Focal Loss for the classification loss ${\text{L}}_{\text{cls}}\;$ to address sample imbalance, especially in scenarios with imbalanced positive and negative samples. The Focal Loss is defined as Equation 10.


(10) $$L_{\text{cls}}=-\sum_{i}{\text{α}}_{t}{\left(1-p_{t}\right)}^{\text{γ}}\log(p_{t})$$

Among them, $p_{t}$ is the model’s predicted probability of the true class, ${\text{α}}_{t}$ is the class weight, which balances the impact of the number of samples in different classes, and γ  is the adjustment factor, which reduces the loss contribution of easy samples and focuses on hard samples. Through Focal Loss, we can reduce the impact of a large number of easy negative samples on the loss, so that the model can pay more attention to hard positive samples.

#### 3.4.3 Design of the regression loss

For the regression loss ${\text{L}}_{\text{reg}}$ , measuring bounding box localization accuracy, we use the Smooth $L_{1}$ Loss as expressed in Equation 11.


(11) $$L_{\text{reg}}(t,v)=\sum_{i\in \{x,y,w,h\}}{\text{smooth}}_{L1}(t_{i}-v_{i})$$

Where ${\text{t}}_{\text{i}}$ is the predicted bounding box parameter, ${\text{v}}_{\text{i}}$ is the truth bounding box parameter, {x,y,w,h} are location information of the box and smooth_L1_ (·) is the Smooth loss function.

#### 3.4.4 Modeling of the feature aggregation loss

To ensure effective utilization of support set information and prevent overfitting or redundancy due to the attention mechanism, we introduce the feature aggregation loss $L_{\text{agg}}$ , consisting of attention regularization and sparsity constraint.

##### 3.4.4.1 Attention regularization

We use Attention Entropy as a regularization term to prevent attention weights from over-concentrating on a few support samples, encouraging comprehensive utilization of support set information.

Attention Weight Matrix, for query set sample *i* and support set sample *j*, the attention weight $a_{ij}$ is computed as Equation 12:


(12) $$a_{ij}=\frac{\exp(s_{ij})}{\sum_{k=1}^{N_{s}}\exp(s_{ik})}$$

where the similarity score $s_{ij}$ is: $s_{ij}=\frac{q_{i}\cdot k_{j}}{\sqrt{d_{k}}}$ , where $q_{i}$ is the query vector of *i* query sample, $k_{j}\;$ is the key vector supporting sample *j*, and $d_{k}$ is the dimension of the key vector.

###### 3.4.4.1.1 Attention entropy regularization term

The attention entropy regularization term is formulated as Equation 13.


(13) $$L_{\text{attn\_reg}}=\frac{1}{N_{q}}\sum_{i=1}^{N_{q}}(-\sum_{j=1}^{N_{s}}a_{ij}\log a_{ij})$$

where $N_{q}$ and $N_{s}$ are the numbers of query and support samples.

By maximizing the attention entropy (i.e., minimizing the negative attention entropy), the attention weights are encouraged to be more evenly distributed over the support set, preventing over-reliance on a small number of support samples.

##### 3.4.4.2 Sparsity constraint

To encourage sparsity in attention weights, focusing on the most relevant support samples and enhancing discriminative power, we impose a sparsity constraint on the unnormalized similarity scores $s_{ij}$ , Sparsity Regularization Term is defined in Equation 14.


(14) $$L_{\text{sparse}}=\frac{1}{N_{q}}\sum_{i=1}^{N_{q}}\sum_{j=1}^{N_{s}}|s_{ij}|$$

By summing the absolute values of the similarity scores, the model is encouraged to generate a sparser similarity matrix, making the attention weights more inclined to a small number of important support samples.

###### 3.4.4.2.1 Complete feature aggregation loss function

Combining the attention regularization Equation 13 and sparsity constraint Equation 14, the feature aggregation loss is computed by Equation 15.


(15) $$L_{\text{agg}}=(\frac{1}{N_{q}}\sum_{i=1}^{N_{q}}\sum_{j=1}^{N_{s}}a_{ij}\log a_{ij})+(\frac{1}{N_{q}}\sum_{i=1}^{N_{q}}\sum_{j=1}^{N_{s}}|s_{ij}|)$$

These regularization terms help the model better utilize the information of the support set and prevent the attention weights from being over-concentrated or over-dispersed, thereby improving the effect of feature aggregation and improving the detection performance of the model.

###### 3.4.4.2.2 The choice of the balance coefficients

${\text{λ}}_{1}$ and ${\text{λ}}_{2}$ has an important impact on the performance of the model. Usually, we can adjust the values of these coefficients through experimental verification to achieve the best performance. In general, the values of ${\text{λ}}_{1}$ and ${\text{λ}}_{2}\;$ can be set to 1 or scaled according to the relative size of the loss terms.

By jointly optimizing the above loss functions, our model can simultaneously achieve the optimization goals of classification accuracy, positioning accuracy, feature representation, and imbalance during training. That is, through Focal Loss and SCL, the model more accurately predicts the target class to improve classification accuracy. By optimizing regression loss, the model can more accurately locate the target boundary to improve positioning accuracy. Through feature aggregation and SCL, the model’s feature expression ability is improved, thereby enhancing feature representation. The weight mechanism introduced in the loss function enables the model to pay more attention to minority classes and hard samples to adapt to unbalanced data.

### 3.5 Training strategies

#### 3.5.1 Multi-task joint training

We employed a multi-task learning approach to facilitate collaborative optimization among various model components. In each training iteration, localization loss, classification loss, feature aggregation loss, and supervised contrastive loss were computed. These losses were then combined into a single cumulative loss $L_{\text{total}}$ . Backpropagation and parameter updates were performed based on this total loss, ensuring joint optimization of all components. This approach promotes better feature representation, faster convergence, and improved generalization by encouraging mutual information sharing among tasks.

#### 3.5.2 Learning rate and optimizer

To stabilize training and prevent initial oscillations, we adopted a piecewise or cosine annealing learning rate decay schedule. This strategy lowers the learning rate in a controlled manner, allowing the model to converge steadily. For the optimizer, we used Stochastic Gradient Descent (SGD) with momentum to accelerate convergence and smooth out gradients. The momentum factor was tuned on the validation set to achieve the best balance between convergence speed and stability.

#### 3.5.3 Weight initialization

To expedite convergence and leverage prior knowledge, we initialized model parameters using ImageNet-pretrained backbone weights. Newly added modules, such as the Feature Aggregation Module (FAM) and the projection head for contrastive learning, were initialized using Kaiming initialization. This approach ensures that important structural components inherit robust feature representations while newly introduced parameters adapt rapidly.

#### 3.5.4 Regularization and overfitting prevention

To mitigate overfitting, we applied L2 regularization (weight decay) in the optimizer. Additionally, dropout was introduced in fully connected layers and within the FAM to stochastically deactivate a fraction of neurons during training. This not only prevents the model from over-relying on specific neurons but also improves its capacity to generalize to unseen data.

#### 3.5.5 Data augmentation

We employed a variety of image augmentation techniques, including random cropping, rotation, flipping, and color jittering, to increase data diversity and reduce overfitting. For class imbalance issues—especially in few-shot scenarios—we performed sample balancing by oversampling minority classes or undersampling majority classes, aiming to achieve a more balanced and representative training set. This augmentation and balancing strategy is particularly critical in 3-shot, 5-shot, and 10-shot experiments, where the training samples are limited.

#### 3.5.6 Training process monitoring

We continuously tracked training progress by observing the loss curves (localization, classification, feature aggregation, and supervised contrastive) to ensure stable convergence. Model performance was periodically evaluated on a validation set using metrics such as mean average precision (mAP) or recall. If the performance began to plateau or degrade, we adjusted hyperparameters—including learning rate, momentum, and regularization factors—accordingly.

#### 3.5.7 Hyperparameter adjustment

In our loss function, the coefficients $\lambda_{1}$ and $\lambda_{2}$ determine the relative importance of each sub-loss. We fine-tuned these values based on validation performance, ensuring that no single loss term dominated the training.

Of particular importance is the temperature parameter τ in the supervised contrastive loss, which controls the smoothness of the probability distribution when computing similarities among samples. Proper tuning of \(\tau\) helps stabilize contrastive learning by balancing the separation between positive and negative pairs. After addressing reviewer concerns, we corrected the temperature parameter usage by referencing the optimal settings reported in the official FSCE(Sun, B et al., 2021) experiment. Few-Shot Settings: For 3-shot, 5-shot, and 10-shot training, the positive sample IoU thresholds were set to 0.6, 0.7, and 0.8, respectively. Temperature Coefficients τ : Consistently set to 0.2 for 3-shot, 5-shot, and 10-shot. Aggregate Loss Weights $\lambda_{1}\;$ and Comparison Loss Weights $\lambda_{2}$ : Set to 0.2, 0.5, and 0.5, respectively, in the 3-shot, 5-shot, and 10-shot scenarios.

#### 3.5.8 Model saving and selection

To safeguard against unexpected interruptions, we regularly saved model checkpoints during training. Each checkpoint contained the model weights, optimizer state, and current learning rate. After completing training, we selected the best-performing checkpoint based on validation metrics for final testing and deployment. This ensures that the model used in downstream tasks represents the most robust and accurate version learned during training.

By implementing the above multi-task joint training strategy with detailed hyperparameter tuning, our model demonstrated stable and efficient training, fully harnessing the benefits of collaborative optimization. The experimental results (presented in Section 4) indicate marked improvements in both convergence speed and overall performance, corroborating the effectiveness of these methodologies. Additionally, fine-tuning in the two-stage Faster R-CNN architecture proved essential for adapting the model to specific datasets and tasks, yielding enhanced robustness and accuracy. This tailored approach ensures alignment with the unique characteristics of real-world applications, thereby solidifying the model’s practical relevance.

## 4 Experiments

### 4.1 Dataset, experimental configuration and parameter settings

This research introduces the PestDet dataset to support a few-shot pest detection method based on feature aggregation and SCL. PestDet, consisting of approximately 82,000 images, integrates data from the IP102 dataset (Wu et al., 2019), the IDADP dataset (Chen and Yuan, 2019), and additional images from the internet and production environments. It includes targets at individual, medium, collective, and mixed levels, covering various pest stages. The IP102 dataset, comprising over 75,000 images of 102 pests, served as the primary source, with 19,000 images containing detailed detection annotations. The IDADP dataset added 4,700 images of typical agricultural pests. Additional samples from tropical regions further enhanced dataset diversity.Dataset preprocessing included cleaning duplicate images using a pre-trained vision transformer (ViT), re-annotating different pest stages, and resolution equalization to balance image resolutions. Annotations were optimized by removing zero-area bounding boxes, duplicate boxes, and correcting incorrect labels. These steps improved dataset quality, ensuring effective training and better detection performance.

To construct the object detection dataset for this study, we leveraged the PestDet dataset, which was originally designed for classification and object detection tasks and includes 102 pest classes labeled from 0 to 101. **Table 2** provides detailed statistics of the PestDet dataset, including the total number of images, the number of bounding boxes, and the number of single-bounding-box images for each pest class. However, the bounding box distribution in PestDet is highly imbalanced, with some classes having significantly more annotations than others, leading to a model bias toward classes with more bounding boxes during training. To address this issue and to focus on FSOD while considering computational constraints, we constructed a balanced subset, PestDet20, by selecting 20 pest classes from PestDet. These classes were chosen to represent pests commonly found in tropical and subtropical economic crops, characterized by individual diversity and complex backgrounds. The selection process, detailed in **Table 1**, involved sorting all classes by bounding box count in descending order, excluding redundant or subset classes (e.g., those with large overlaps between larvae and adult forms of the same pest), and finally selecting the top 20 classes based on bounding box count. The selected classes are numbered {0, 3, 14, 15, 16, 21, 24, 25, 26, 37, 39, 48, 50, 66, 67, 70, 76, 95, 99, 101}, following the original PestDet numbering. Inspired by the 20-class structure of the PASCAL VOC dataset as outlined in the TFA standard, the PestDet20 dataset was constructed to provide a balanced and representative foundation for addressing the unique challenges of FSOD in pest management.

**Table 2:** Overall class image information of training set and test set.

Metrics	Number of images	Number of total annotated boxes	Number of images with unique annotated boxes
mean	187	217	174
std	343	362	334
min	3	5	2
25%	44	53	31
50%	92.50	120	83
75%	183	243	175
max	2859	2896	2826

**Figure 2:**
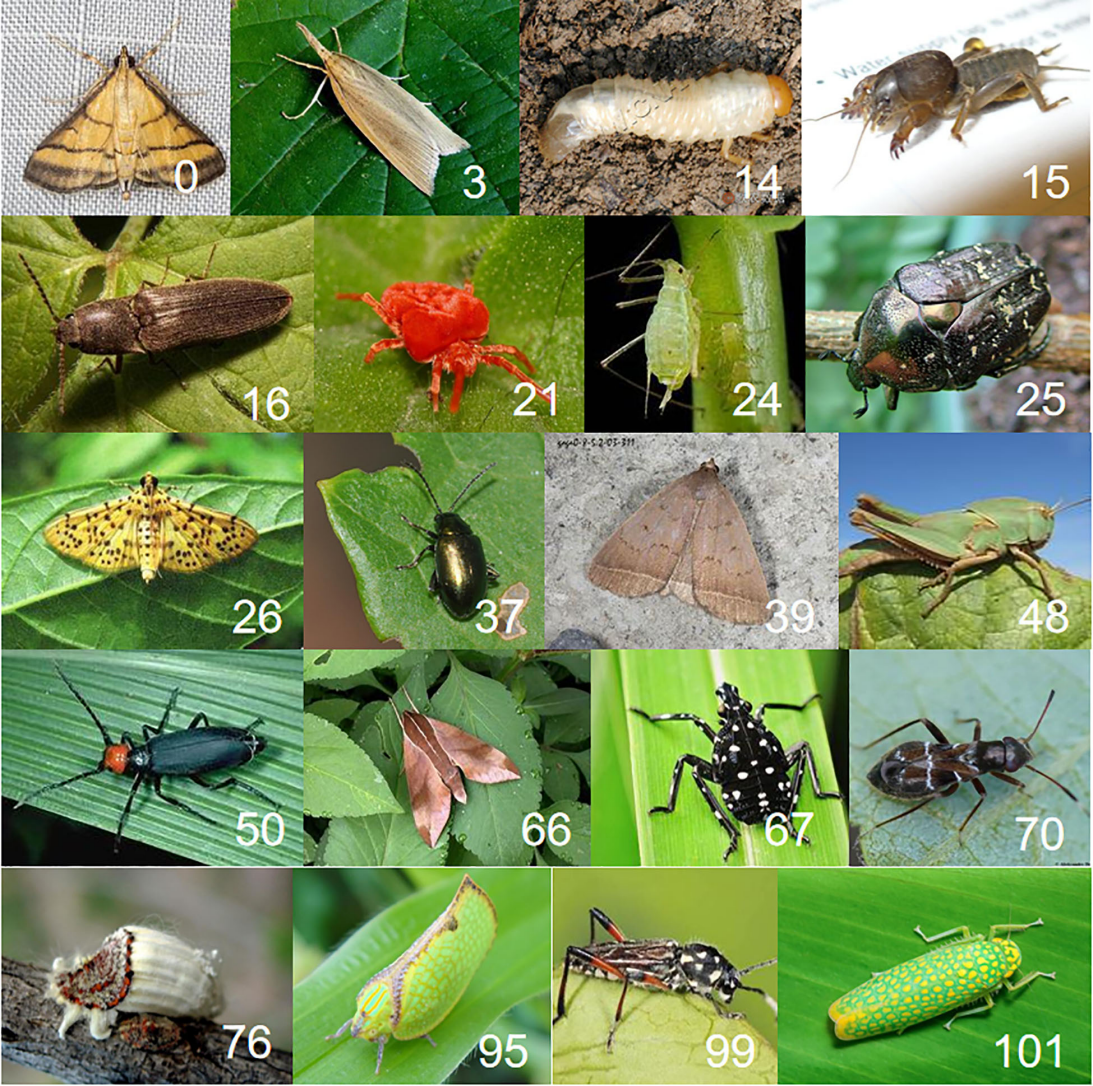
Examples of all classes of pests in the dataset.

To facilitate analysis and experimentation, a few-shot pest dataset, PestDet20, was created according to selected standards. Class statistics are summarized in **Table 3**. The training set includes 5,076 images with 5,590 bounding boxes, while the testing set has 1,177 images and 1,292 bounding boxes, split by the typical 8:2 ratio. **Figure 2** presents examples of the 20 pest classes studied. In the fine-tuning-based few-shot object detection task, the model training and testing process is divided into two stages: the base stage and the fine-tuning stage. The base stage consists of training and testing, where the training phase uses all samples of the base classes from the training set, and the testing phase uses all samples of the base classes from the testing set. Similarly, the fine-tuning stage also consists of training and testing. During the training phase of the fine-tuning stage, 3, 5, or 10 samples from both the base classes and the novel classes in the training set are used. For testing in the fine-tuning stage, all samples from both the base classes and the novel classes in the testing set are used.

**Table 3:** Image information of 20 selected pests.

Class number	Name	Number of training set images	Number of test set images	Number of training set annotation boxes	Number of test set annotation boxes
0	Rice leaf roller	131	34	141	38
3	Rice stem borer	126	33	138	34
14	Grub	331	80	532	108
15	Mole cricket	400	80	400	80
16	Wireworm	325	80	405	104
21	Red spider	125	31	128	36
24	Aphid	400	80	400	80
25	White-spotted flower beetle	145	40	173	46
26	Peach borer	188	45	199	47
37	Flea beetle	253	65	285	70
39	Beet armyworm	317	81	322	81
48	Acridoidea	400	80	400	80
50	Blister beetle	338	85	366	94
66	Grape hawkmoth	197	53	197	53
67	Cicada	253	63	253	63
70	Lycophoridae	400	80	400	80
76	Cotton scale	121	28	216	55
95	Brown-margined moth	134	33	142	36
99	Spine-chested longhorn beetle	92	26	93	27
101	Cicadidae	400	80	400	80
Total		5076	1177	5590	1292

Using the feature aggregation-based fine-tuning method from VFA (Han et al., 2023), the FSOD dataset was divided with a random shuffling strategy. The 20 selected pest classes {0, 3, 14, 15, 16, 21, 24, 25, 26, 37, 39, 48, 50, 66, 67, 70, 76, 95, 99, 101} were shuffled three times, creating distinct class arrays. In each shuffle, 15 classes served as base classes, while the remaining 5 were designated as novel classes, as shown in **Table 4**.

**Table 4:** Classification.

Split	All classes	Basic classes	New classes
1	{15, 76, 24, 39, 14, 67, 16, 95, 25, 3, 66, 0, 101, 99, 37, 70, 26, 50, 48, 21}	{15, 76, 24, 39, 14, 67, 16, 95, 25, 3, 66, 0, 101, 99, 37}	{70, 26, 50, 48, 21}
2	{101, 14, 48, 15, 3, 67, 39, 66, 76, 50, 95, 26, 37, 24, 0, 16, 21, 99, 70, 25}	{101, 14, 48, 15, 3, 67, 39, 66, 76, 50, 95, 26, 37, 24, 0}	{16, 21, 99, 70, 25}
3	{0, 48, 14, 99, 3, 21, 39, 66, 16, 37, 50, 26, 25, 70, 24, 67, 101, 76, 15, 95}	{0, 48, 14, 99, 3, 21, 39, 66, 16, 37, 50, 26, 25, 70, 24}	{67, 101, 76, 15, 95}

The training set, used as the support set, and the testing set, used as the query set, evaluated the model’s stability and robustness. Strong performance across subsets indicates model stability, while poor performance on certain subsets suggests sensitivity to specific classes or features. After dividing the base classes and novel classes, we trained and tested the model using 30 random seeds and obtained the average results to compare with methods that use random seeds. For the fine-tuning phase, we sampled images from each class to construct the training set, with the number of sampled images set to 3, 5, and 10, respectively. This approach ensures that the sample sizes of base classes and novel classes during the fine-tuning phase are balanced, thereby reducing the model’s bias toward the base classes.

#### 4.1.1 Experimental configuration and parameter settings

Compared to few-shot classification and regular object detection tasks, FSOD faces more challenges. Its training dataset is mainly divided into two classes: base classes, with abundant annotated data, and novel classes, with limited annotated data. The main goal of FSOD is to significantly improve detection performance for novel classes while maintaining high detection accuracy for base class. FSOD effectively reduces the dependence of object detection models on large amounts of training data, solves the problem of imbalanced annotations in training data, and has significant practical value and a wide range of applications.

This study compared three classic FSOD algorithms: YOLO (Khanam and Hussain, 2024), TFA (Wang et al., 2020) VFAr43 (Han et al., 2023) and FSCE (Sun et al., 2021). Experiments were conducted on the Ubuntu operating system, using Python as the main development language, based on the PyTorch deep learning framework, with mmfewshot used for FSOD model training and testing. The hardware environment included two NVIDIA GeForce RTX 4090 GPUs with 24G VRAM each, an Intel(R) Xeon(R) CPU E5–2680 v3, and 64G of memory.

In experimental hyperparameter settings, SGD was selected as the optimizer, with an initial learning rate of 0.02, a batch size of 4, and 18,000 training iterations, with model evaluation intervals of 3,000 iterations. During the fine-tuning stage, the learning rate was adjusted to 0.001, and iteration numbers and evaluation intervals were adjusted according to different novel classes. During 3-shot, 5-shot, and 10-shot training, the IoU threshold for positive samples was set to 0.6, 0.7, and 0.8, respectively, the temperature coefficient was set to 0.2, and contrastive loss weights were set to 0.2, 0.5, and 0.5 respectively.

### 4.2 Evaluation indicators

#### 4.2.1 Evaluation criteria


(16) $$precision=\frac{TP}{TP+FP}$$

(17) $$recall=\frac{TP}{TP+FN}$$

TP , FP , and FN represent true positive, false positive, and false negative, respectively. Precision and recall are defined as Equations 16, 17, respectively.

When the sum of IoU between the predicted box and the target box exceeds 0.5, the predicted box is positive, otherwise it is negative.


(18) $$AP=\int_{0}^{1}p(r)d_{r}$$

*AP* represents the area below the precision-recall curve, calculated as shown in Equation 18, with accuracy as the ordinate and recall as the abscissa.

In FSOD, base class performance is typically measured using bAP, while nAP is used to assess the performance of novel classes. Suppose class $i(i=1,2,\ldots ,N_{B})$ belongs to base classes, and class $j(j=N_{B}+1,N_{B}+2,\ldots ,N)$ belongs to novel classes (*N* denotes the number of the training classes), bAP and nAP can be expressed by Equations 19, 20.


(19) $$bAP=\frac{1}{N_{B}}\sum_{i=1}^{N_{B}}AP_{i}$$

(20) $$nAP=\frac{1}{N-N_{B}}\sum_{j=N_{B}+1}^{N}AP_{j}$$

In the subsequent analysis, we also utilize mAP, expressed by Equation 21.


(21) $$mAP=\frac{\sum_{c=1}^{n}AP_{c}}{n}$$

where *c* represents the class, *n* represents the number of classes, and mAP represents the average AP of multiple classes. The overall effect of multi-class target detection can be represented by mAP .

### 4.3 Comparative analysis of experiments

Our method will be compared with several classic FSOD methods, including the classic fine-tuning method TFA (Wang et al., 2020), the feature aggregation method based on the meta-learning framework VFA (Han et al., 2023), and the two-stage learning method (Sun et al., 2021) based on contrastive learning. Additionally, we incorporate YOLO (Khanam and Hussain, 2024), a widely adopted one-stage object detection framework that is particularly known for its real-time performance in various detection tasks. YOLO (You Only Look Once) significantly differs from two-stage models like Faster R-CNN by integrating region proposal and classification into a single, unified network, making it highly efficient and fast for both training and inference. All comparative experiments are trained and tested on the MMFewShot framework produced by Open MMLab. Our model’s indicators are significantly better than most of the most advanced SOTA methods.

#### 4.3.1 Analysis of basic stage results

**Figure 3:**
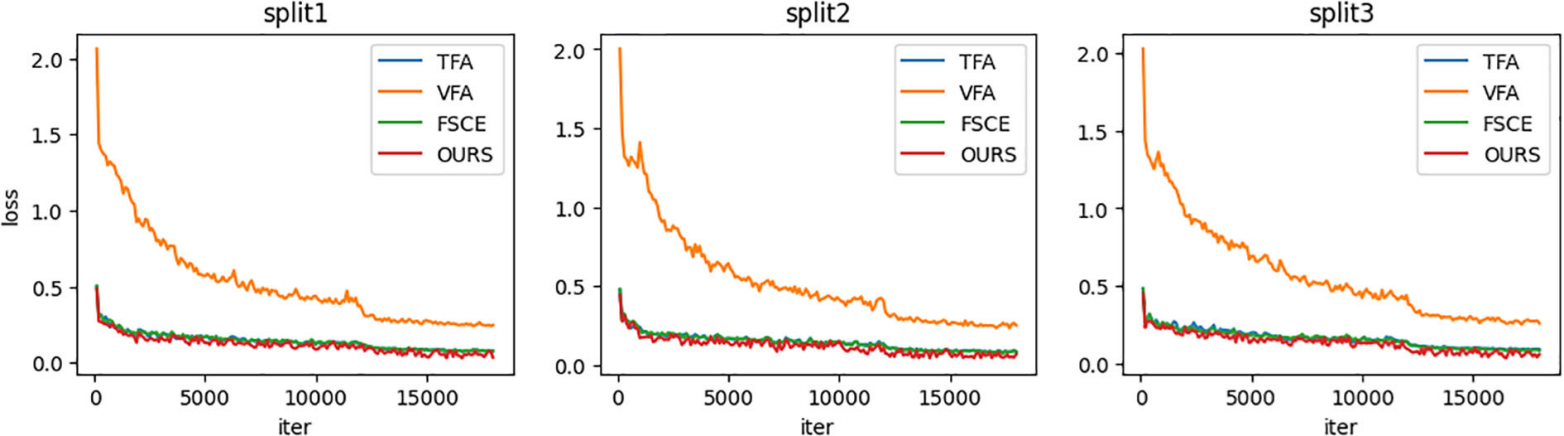
Line chart of overall loss of basic stage training.

**Figure 3** shows the overall loss curves of the three class split sets (split 1, 2, 3) during basic stage training. As can be seen from the figure, the loss curves of TFA, FSCE and **the proposed method (OURS)** are almost completely overlapped, indicating that the learning process of the three methods in the basic training stage is very similar. Since the variational autoencoder introduces additional loss terms during training, the loss of VFA is higher. Overall, the loss of the four methods is gradually decreasing with the increase in the number of training iterations, indicating that the model is constantly learning and improving.

**Figure 4:**
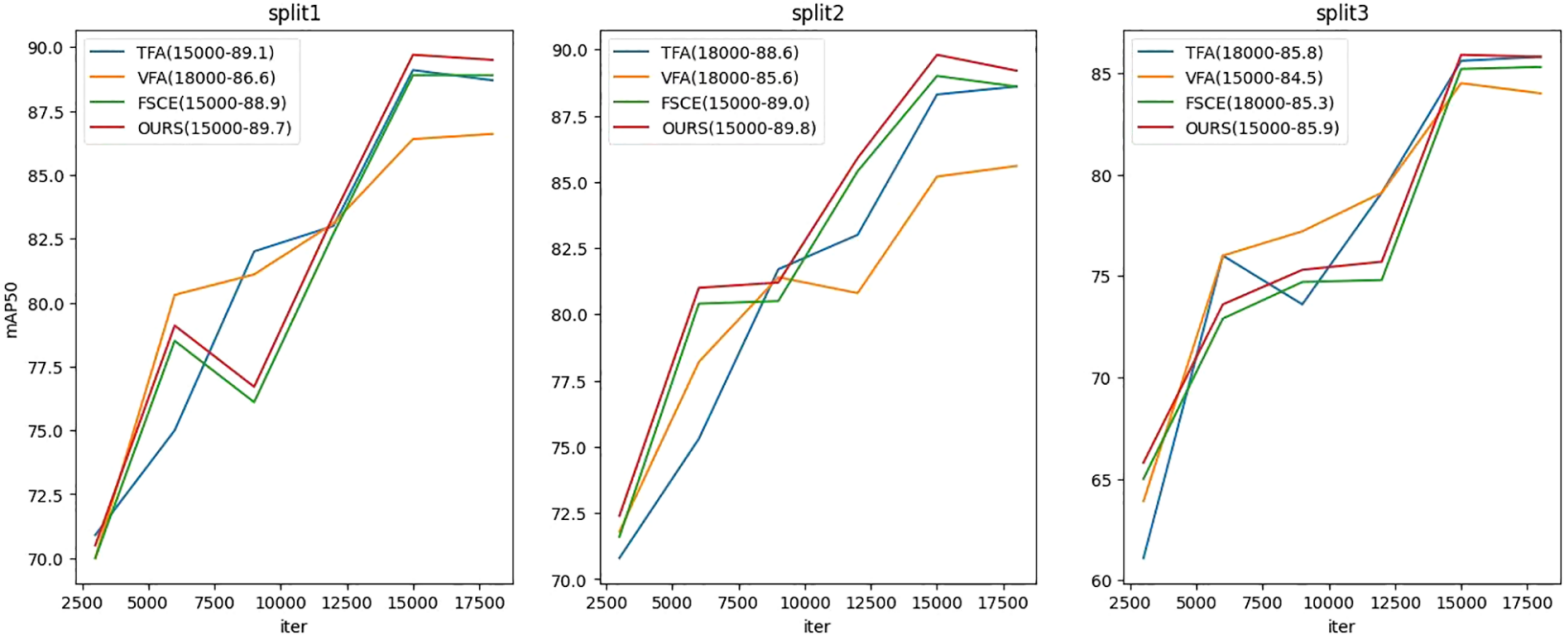
Basic stage testing mAP50 indicator line chart.

**Figure 4** shows the changes in mAP50 (average accuracy under IoU 0.5) of four different class splits during basic stage, and the test set is evaluated every 3000 iterations. In split1, VFA, FSCE, and the proposed method reach maximum mAP50 values of 86.6, 88.9, and 89.7 at 18,000 iterations, respectively; while TFA reaches a maximum mAP50 value of 89.1 at 15,000 iterations, but drops at 18,000 iterations, indicating possible overfitting. In split2, TFA and VFA reach maximum mAP50 values ​​of 88.6 and 85.6 at 18,000 iterations, respectively, while FSCE and the proposed method reach 89.0 and 89.8 at 15,000 iterations, and also show overfitting at 18,000 iterations. In split3, TFA and FSCE reached 85.8 and 85.3 respectively at 18,000 iterations, while VFA and the proposed method reached the maximum value of 84.5 and 85.9 at 15,000 iterations, but overfitting also occurred at 18,000 iterations. These results reflect the differences in the sensitivity of different methods to the number of training iterations and the stability in the later stages of training.

Based on these results, we will adopt the following strategy for subsequent fine-tuning: selecting the models saved at the point where mAP50 achieves the highest value in splits 1 to 3 as the starting point for fine-tuning. the proposed method is to leverage the model state that achieves optimal performance during the base stage to further enhance its performance in the few-shot object detection task.

#### 4.3.2 Fine-tuning experimental results analysis

**Figure 5:**
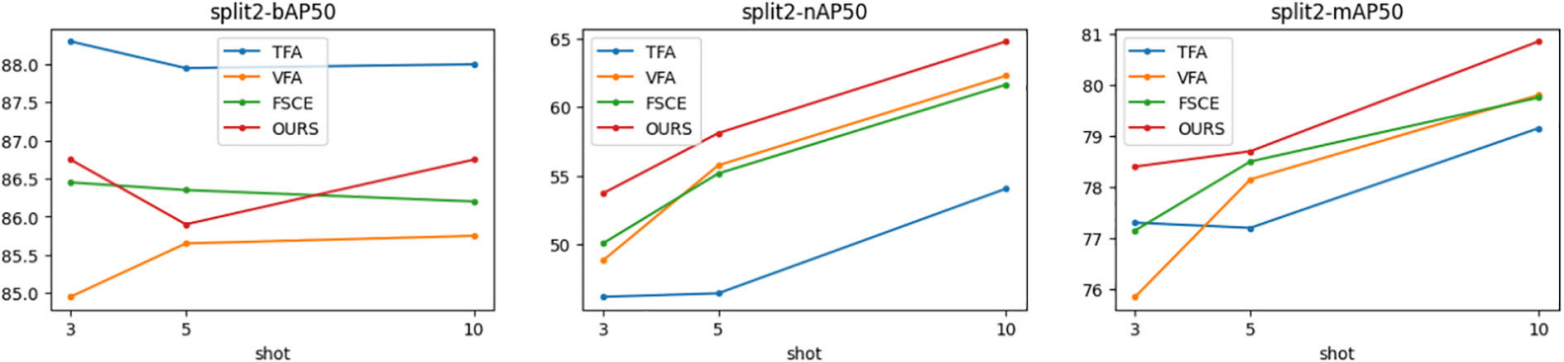
Trends of bAP50, nAP50, and mAP50 for split1.

**Figure 6:**
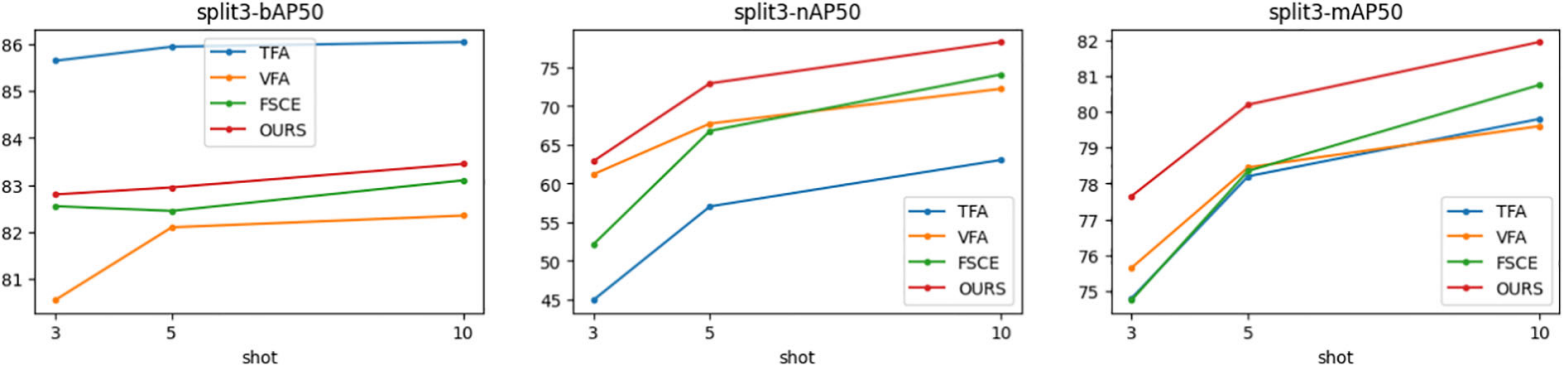
Trends of bAP50, nAP50, and mAP50 for split2.

**Figure 7:**
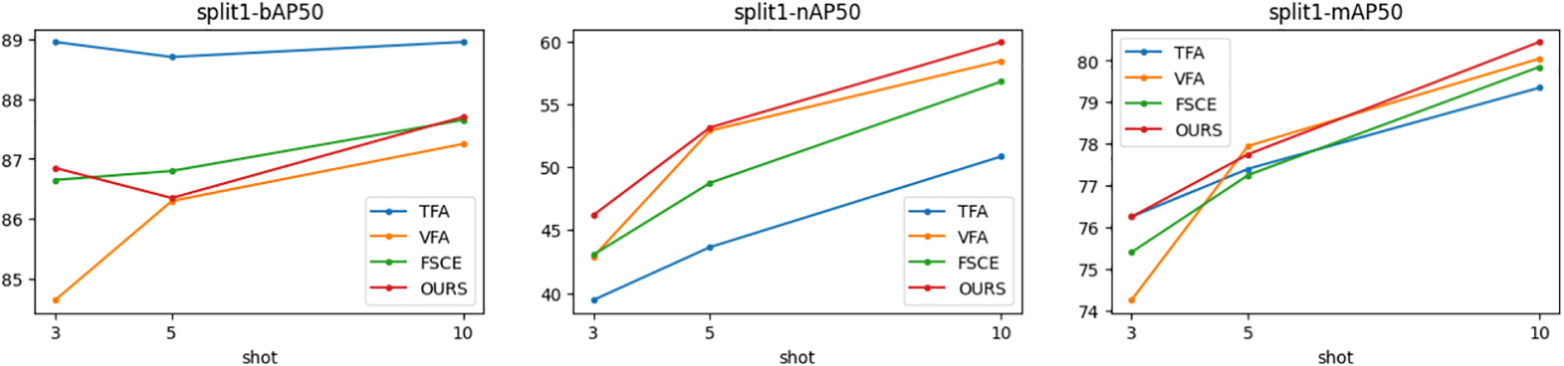
Trends of bAP50, nAP50 and mAP50 for split3.

**Figures 5**–**7** show the visualization results of the relevant data after two rounds of random sampling and fine-tuning, and the results on the test set with different sample numbers (3, 5, and 10), covering splits 1, 2, and 3. The performance of each method (TFA, VFA, FSCE, and the proposed method) is measured by the average precision of the base class (bAP50), the average precision of the new class (nAP50), and the overall average precision (mAP50).As can be seen from **Figures 5**–**7**, TFA and VFA show an inverse relationship in performance: TFA performs well on the base class (bAP50), but is relatively weak on the new class (nAP50), which indicates that TFA may not be able to effectively transfer knowledge to the new class. In contrast, VFA performs well in the new class but poorly in the base class, which indicates that the model may sacrifice the performance of the base class to adapt to the new class. In contrast, FSCE performs evenly in the two classes and shows better robustness. the proposed method performs better on the basis of FSCE. Under certain split and shot configurations, the proposed method even slightly outperforms VFA in terms of new classes and overall accuracy, indicating its excellent ability in balancing the performance difference between base and new classes.

In the nAP50 graph of new classes for split3, VFA outperforms other methods under 3-shot conditions; but its performance improves only slightly with the increase in sample size, increasing by only 11.04% from 3-shot to 10-shot. In contrast, the performance of the proposed method improves significantly, increasing by 23.95% from 3-shot to 10-shot. To further study this phenomenon, a third random sampling fine-tuning training experiment was conducted based on the split3 dataset.

**Figure 7** shows the changing trends of bAP50 and nAP50 during split3 fine-tuning training under different shot conditions. The performance of FSCE and the proposed method in bAP50 is always between TFA and VFA, but its maximum nAP50 exceeds that of other methods, which highlights the advantage of the proposed method in balancing the performance of base and new classes.

To comprehensively compare the performance of detection methods across 20 tropical pest classes, we included the YOLO model (specifically the YOLO11x version) as a benchmark for FSOD tasks. **Table 5** and relevant results incorporate the YOLO method alongside TFA, VFA, FSCE, and the proposed method (OURS). While YOLO is known for its efficiency in real-time detection tasks due to lower computational complexity, the results indicate that this advantage does not translate into better performance in FSOD scenarios. The results show that YOLO, TFA, VFA, FSCE and the proposed method all show high AP values ​​under 3-shot, 5-shot and 10-shot conditions, proving the stability of the methods. With the increase of sample size, the performance of the three methods in new classes gradually improves, especially when the sample size is small, the detection performance is significantly improved with a slight increase in sample size.

**Table 5:** AP values and mAP values of four methods for detecting 20 types of pests.

Cat.	Num.	YOLO11-FSOD			TFA			VFA			FSCE			OURS		
		3shot	5shot	10shot	3shot	5shot	10shot	3shot	5shot	10shot	3shot	5shot	10shot	3shot	5shot	10shot
Base class AP	0	82.5	84.4	87	96	96	95.8	90.2	97.9	90.4	91.7	92.1	92	90.7	89.8	96.2
	48	59.6	61.2	57.9	68.5	69	69.8	63.4	67	69.2	65.6	68	66.6	67	66.7	63.6
	14	83	89.7	86.2	95.4	92.9	89.5	88.4	88.7	88.5	95.9	94.5	88	89.2	87.7	88.3
	99	80.8	75.3	68.9	85.2	85	84.8	86.8	88.2	89.1	75.4	77.5	82.5	78.4	81.7	76.7
	3	85.9	77	79.3	69.6	71.3	71.4	72.6	77.4	75	66.3	74.3	71.5	69.9	76.1	80.3
	21	77.6	80.5	83.8	89	88.8	88.8	74.7	72.8	73.7	85.3	80.9	81	87.8	87.3	86.7
	39	84.8	76.4	79.6	88	88	93.3	88.2	88.4	89.3	83.8	85.7	86.6	89.8	89.1	89.3
	66	95.1	96.4	97.8	89.7	90	89.9	89.1	89.1	89.1	92.9	93.5	89.9	94.9	90.4	90.7
	16	67.2	72.8	68.9	84.3	84.4	80.9	67.7	66.5	70.7	78.8	80.2	79.4	81.4	79.3	83.8
	37	79.2	77.4	71.8	99.4	99.3	97.8	88	88.1	88.4	89	89.6	93.8	88.9	89.1	90.4
	50	82.8	73.8	80.7	85.1	85.5	86.1	86.5	87.4	88.1	83	84.1	84.5	83.8	79.1	85.9
	26	80.7	80	81.6	84.6	83.7	83.1	66.6	71.6	78.6	80.3	77.7	78.8	84.8	80.4	85.7
	25	82.8	83.3	86.4	89	89.3	89.2	81.8	84.6	88.8	89.2	89.3	88.4	87.9	88.6	90.5
	70	63.2	54.4	57.9	67.8	67.7	66.1	52.8	59.3	66.2	68.8	73.2	71.5	73.6	70.6	62.8
	24	85.8	75.4	79.5	87.6	86.7	87.7	84.9	84.5	84.5	78.4	76.5	83.8	84.8	84.1	85.7
New class AP	67	72.7	98.2	98.5	81.8	82.7	83.3	92.1	94	96.5	90.9	90.1	94.4	90.9	95	97.6
	101	63.9	81.6	88.8	17	25.7	49	38.5	55.3	67.7	18.7	36.9	66.7	35.9	65	82.5
	76	17.3	23.8	36.8	12	27.8	28.8	17.2	28.8	33.7	28.3	33.1	34.6	30.1	36.8	44.9
	15	51.4	85.9	89.4	65	64.8	75.2	66.6	83.2	88.3	68.9	79.8	89.7	69	77.9	87.7
	95	60.5	79.3	72.4	51.2	58.6	64.4	42.5	38.4	57.4	67.5	68.1	72.8	74.1	76.1	83.5
mAP	Base class	79.4	77.2	77.8	85.2	85.1	84.9	78.7	80.7	84.9	81.6	82.4	82.5	83.5	82.6	83.7
	New class	53.2	73.8	77.2	45.3	51.9	60.1	51.3	59.9	60.1	54.8	61.8	71.6	60	70.1	79.2
	All class	66.3	75.5	77.5	75.3	76.9	78.7	71.9	75.6	78.7	74.9	77.2	79.8	75.9	79.5	82.6

In terms of detection performance in each class, the proposed method shows an upward or stable trend in mAP value with the increase of sample size, while YOLO, TFA, VFA and FSCE have certain fluctuations. Especially in the new class, the proposed method achieved the maximum mAP value of 82% in the 10-shot experiment, which is significantly better than YOLO, TFA, VFA and FSCE. In addition, the proposed method shows particularly excellent performance in specific classes such as 15 and 95, and significantly improves AP in the challenging 101 class (Cicadellidae). Compared with other methods, its mAP value is nearly 3 times higher, reflecting the powerful feature aggregation and migration capabilities of the proposed method.

**Figure 8:**
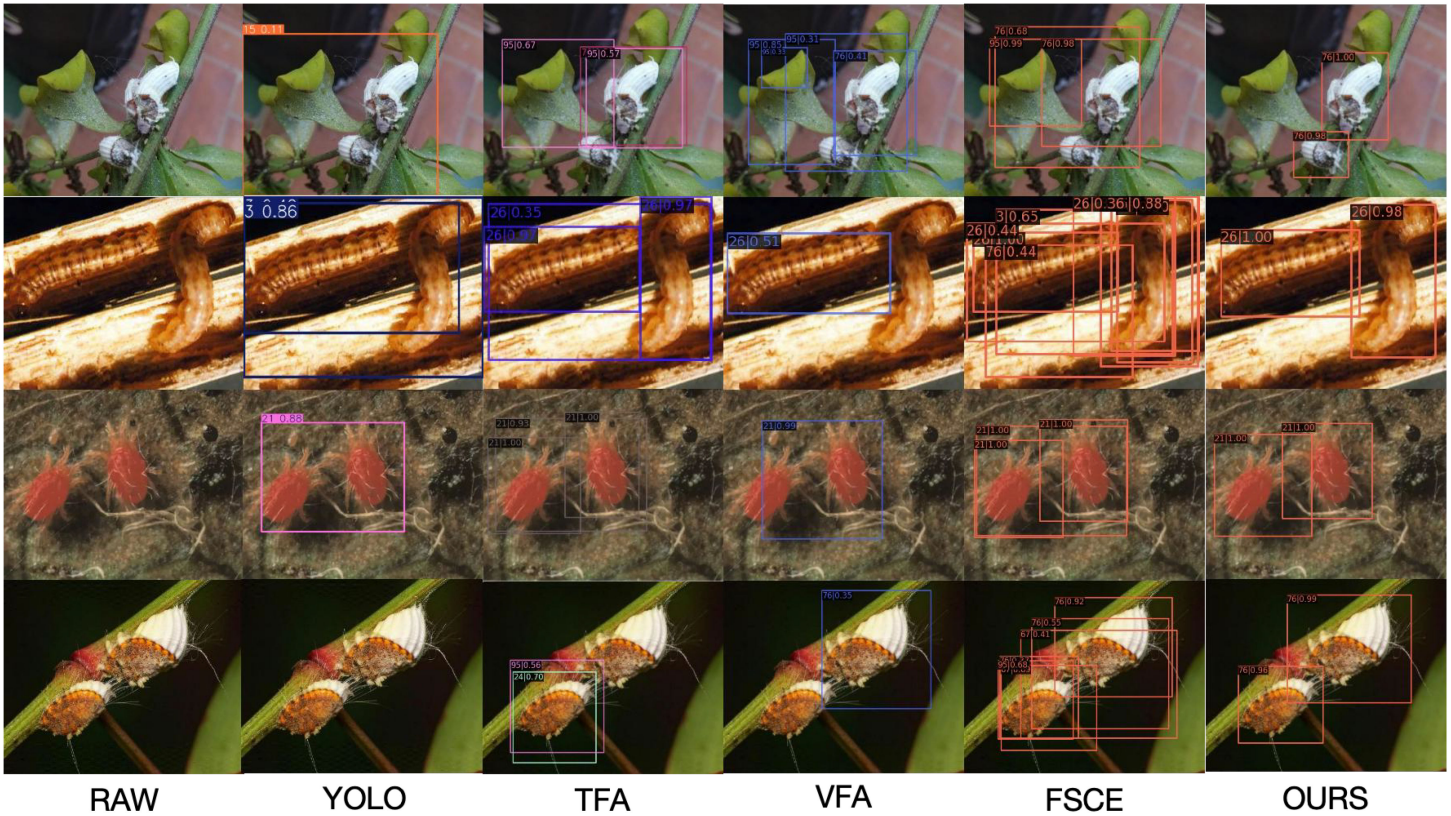
Visual comparison of original image RAW, YOLO, TFA, VFA, FSCE and OURS.

Although the mAP values ​​of most classes are above 70, indicating that the proposed method can effectively detect these pests, the mAP values ​​of other methods are relatively low for classes such as 48, 101, 76, and 95. As can be seen from the relevant images in **Figure 8**, the visual features of these pests are highly similar to the background, or have features that are difficult to distinguish from other classes, making it difficult for YOLO, TFA, VFA, and FSCE methods to accurately identify them. Overall, the mAP value of the proposed method in the new class is nearly 10 percentage points higher than that of YOLO, TFA, VFA, and FSCE on average, showing its significant advantage in the tropical pest detection task.

In terms of detection performance under 3-shot, 5-shot, and 10-shot conditions, YOLO demonstrates relatively lower AP values compared to the other methods. For example, in the 10-shot experiment, YOLO achieves an mAP of 77.5%, whereas the proposed method achieves a significantly higher mAP of 82.6%. Notably, in challenging classes such as 48 and 101, YOLO struggles to distinguish pests with features similar to the background, resulting in mAP values below 60%, significantly lower than the corresponding performance of the proposed method. Overall, while YOLO provides a computationally efficient solution, the trade-off between speed and accuracy limits its applicability in FSOD tasks that prioritize precise detection over real-time processing. The proposed method strikes a better balance by achieving state-of-the-art detection performance, justifying the slightly higher computational cost for critical applications like pest management in tropical agricultural settings.

**Figure 9:**
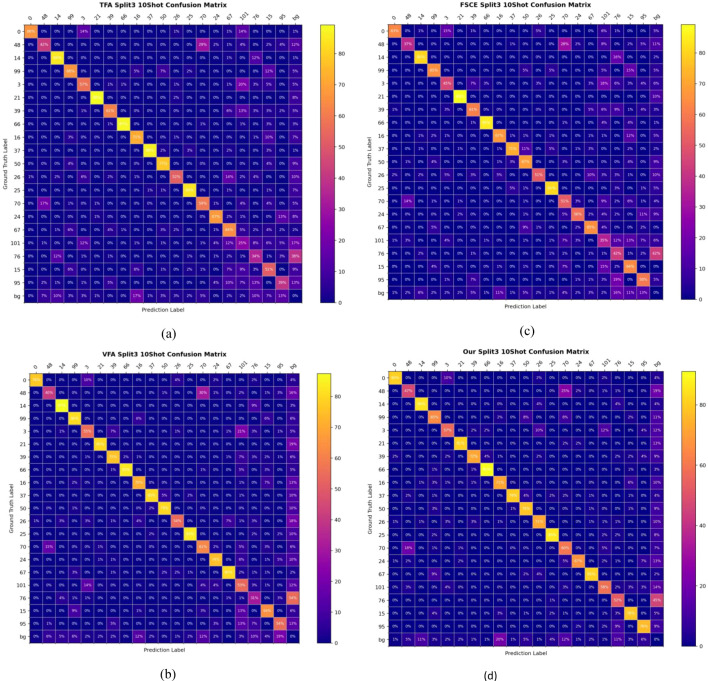
Confusion matrix of split3-10shot for TFA **(a)**, VFA **(b)**, FSCE **(c)** and OURS **(d)**.

As shown in **Figure 9**, from the 10-shot confusion matrix analysis of TFA, VFA, FSCE and the proposed method in split3, the proposed method has obvious advantages in terms of accuracy, missed detection rate and recall rate. First, in terms of accuracy, the proposed method presents higher values ​​on the diagonal, indicating that the model has higher classification accuracy on multiple classes. In contrast, TFA and FSCE methods have lower diagonal accuracy in some classes, showing that the recognition of some classes is not accurate enough under few-sample conditions. In particular, the TFA method has serious misclassification in some classes, while the proposed method is relatively balanced in overall accuracy. In addition, VFA has some misclassification in the background class, while the proposed method is better at distinguishing between targets and backgrounds. Secondly, in terms of missed detection rate performance, the off-diagonal misclassification rate of the proposed method is lower, which means that it has fewer missed detections. In contrast, the FSCE and VFA methods have high missed detection rates in some classes, especially between difficult-to-distinguish classes, which are prone to prediction deviation. FSCE has more obvious misclassification in medium-complexity classes, while VFA shows a tendency to misdetect when the background interference is strong, resulting in an increase in missed detection rate. the proposed method significantly reduces the missed detection rate and improves overall reliability by improving feature extraction. Finally, in terms of recall rate, the proposed method has a higher recall rate in most classes. With fewer misclassifications, the proposed method can effectively identify more real samples, especially in complex backgrounds or with few samples, and the recall performance is more stable. In contrast, the recall rate of the TFA method is low, and it is easy to make recognition errors when the class boundaries are blurred. The recall rate of FSCE is also slightly insufficient when dealing with some subdivided classes.

In summary, the proposed method is superior to other methods in accuracy, missed detection rate and recall rate. Its advantages lie in better feature extraction ability, lower misclassification rate and higher recall rate, making it a more robust model in the case of few samples and complex backgrounds. These improvements enable the proposed method to perform better classification results in the split3 10-shot scenario.

#### 4.3.3 Ablation experiment analysis

**Figure 10:**
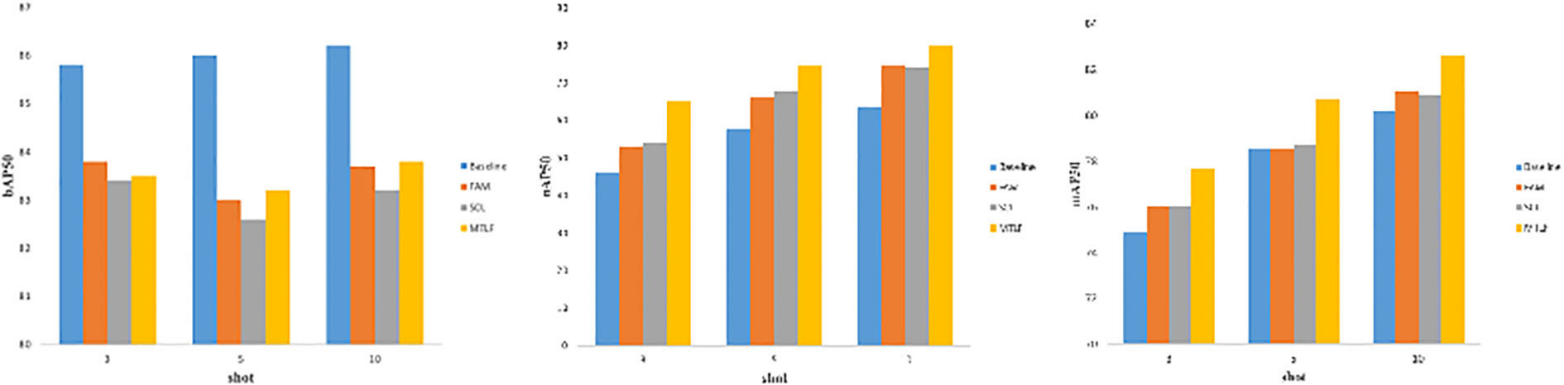
Ablation experiment.

We have evaluated the effectiveness of the modules used in the study, such as the feature aggregation module(FAM), the SCL module SCL, and the multi-task loss optimization MTLF, in detail through ablation experiments. In the ablation study show in **Figure 10**, we systematically introduced three key modules based on the baseline method TFA. By incorporating these modules into the baseline separately, we conducted 3-shot, 5-shot, and 10-shot experiments in the few-shot scenario, and evaluated them in terms of bAP50, nAP50, and mAP50. Through this comprehensive evaluation, we can thoroughly investigate and verify the effectiveness of each component in the framework, which helps to further fully understand the proposed method. The ablation results are shown in **Figure 10**, showing the effect of the key modules. The performance is significantly improved by about 1.1% by introducing FAM alone. Specifically, mAP increases from 0.741 to 0.751 in the case of 3 shots, from 0.772 to 0.779 in the case of 5 shots, and from 0.794 to 0.805 in the case of 10 shots. In addition, the inclusion of the SCL module alone can improve its performance by about 1.5%. In the case of 3 shots, mAP increases from 0.741 to 0.753, in the case of 5 shots, from 0.772 to 0.787, and in the case of 10 shots, from 0.794 to 0.809, highlighting the effectiveness of the SCL module in addressing the multi-scale challenges encountered in pest object detection. In addition, adopting the multi-task loss optimization module as a standalone ensemble on the baseline improves the results by about 3.5%. This improvement is evident in the case of 3 shots, where mAP increases from 0.741 to 0.776, in the case of 5 shots, from 0.772 to 0.807, and in the case of 10 shots, from 0.794 to 0.826.

**Figure 8** shows an example of the comparison of the new class detection results of the proposed method with those of TFA, VFA, and FSCE methods in the dataset. As shown in **Figure 8**, most of the new class objects are correctly detected, demonstrating the efficiency of our model. Other methods have difficulty in effectively detecting new class multi-target situations. In **Figure 5**, we can see that although the insect is similar to the background, our model correctly identifies the background and does not misidentify the insect. Similarly, although there are multiple insect targets in the image, our model can still correctly identify all the targets. Our model can effectively handle size variations and multiple targets, and correctly identify single targets and multiple targets of different sizes. Edge cases, such as overlapping pests or those camouflaged within cluttered backgrounds, posed challenges for all tested models. While the proposed method outperformed others in these scenarios, future work could explore adaptive feature learning techniques or advanced data preprocessing to further improve performance in such cases”.

#### 4.3.4 Model statistical characteristics analysis

To evaluate the stability and differences of the proposed method compared to other methods, statistical analysis and significance tests were conducted. As shown in **Table 6**, our method outperforms the comparison methods (TFA, VFA, and FSCE) in terms of statistical metrics such as mean (Mean), standard deviation (Std), and confidence interval (CI). The mean value of OURS is 79.13, which is higher than TFA (75.30), VFA (75.07), and FSCE (77.26), indicating its superior overall performance. Furthermore, the standard deviation of the proposed method is 1.920, lower than those of VFA (2.471) and FSCE (2.233), demonstrating greater stability. Within the 95% confidence interval, the proposed method exhibits a range of (78.178, 80.088), which is significantly higher than the intervals of other methods, such as TFA (74.541, 76.059). This indicates that the proposed method holds a clear statistical advantage.

**Table 6:** Mean, standard deviation and confidence interval statistical analysis.

Model	Mean	Standard deviation (Std)	Standard error (SE)	Margin of error (MOE)	95% Confidence interval (CI)
TFA	75.30	1.526	0.36	0.759	(74.541, 76.059)
FSCE	77.26	2.233	0.526	1.111	(76.151, 78.372)
VFA	75.07	2.471	0.583	1.229	(73.843, 76.301)
OURS	79.13	1.92	0.453	0.955	(78.178, 80.088)

As presented in **Table 7**, statistical significance tests based on multiple independent experimental results further confirm the advantages of the proposed method compared to TFA, VFA, and FSCE. Using independent t-tests at a significance level of 0.05, the results show that the p-value for the proposed method versus TFA is 0.00116, versus FSCE is 0.03284, and versus VFA is 0.00288—all below 0.05. This demonstrates that the performance of the proposed method is statistically significantly different from the other models. Additionally, the mean value of OURS is 79.13, which is higher than TFA (77.05), FSCE (77.62), and VFA (76.87). These results indicate that the proposed method not only outperforms other models in overall performance but also achieves statistically significant differences across multiple experiments. In summary, the proposed method demonstrates superior stability and performance compared to other models, highlighting its statistical advantages.

**Table 7:** Independent t-test method significance verification analysis.

Model	Comparison model	Significance level	P-value	Model mean	Comparison model mean
OURS	TFA	0.05	0.00116	79.13	77.05
OURS	FSCE	0.05	0.03284	79.13	77.62
OURS	VFA	0.05	0.00288	79.13	76.87

#### 4.3.5 Computational cost and performance trade-off analysis

The analysis of computational complexity and detection performance highlights the trade-offs made in this study. YOLO, known for its efficiency in real-time detection tasks, achieves the lowest computational complexity with 114.5 GFLOPs and relatively moderate mAP values (66.3, 75.5, 77.5 for 3-shot, 5-shot, and 10-shot tasks, respectively). In contrast, OURS, a model based on the Faster R-CNN framework with enhancements such as FAM and SCL, achieves the highest mAP values across all settings (75.9, 79.5, 82.6) at a slightly higher computational cost of 130.2 GFLOPs. These results, summarized in **Table 8**, clearly demonstrate the performance and computational trade-offs between YOLO and OURS. This demonstrates that OURS leverages the computational resources to achieve significant performance gains, particularly in few-shot detection tasks, where accuracy and robustness are critical. While YOLO is more suitable for real-time applications, its lower performance in few-shot tasks highlights its limitations in capturing fine-grained and diverse pest characteristics. Models like TFA, FSCE, and VFA strike a balance between complexity and performance, but they fall short of the proposed method in overall accuracy.

**Table 8:** Model size, computational cost, and performance analysis.

Model	Number of parameters	Calculate costs (GPLOPs)	3shot all class	5shot all class	10shot all class
YOLO11x-FSOD	53.9M	114.5	66.3	75.5	77.5
TFA	60.4M	119.6	75.3	76.9	78.7
FSCE	61.6M	120.8	71.9	75.6	78.7
VFA	68.5M	128.8	74.9	77.2	79.8
Ours	68.1M	130.2	75.9	79.5	82.6

By choosing Faster R-CNN as the base framework, this study prioritizes higher detection accuracy over real-time speed, a trade-off that is justified for applications requiring precise pest management. This approach demonstrates that slight increases in computational complexity are acceptable to achieve substantial performance improvements, aligning with the study’s goal of advancing few-shot object detection in complex agricultural environments.

#### 4.3.6 Practical application and field validation

The proposed algorithm has been integrated into a practical pest management system, whose architectural design (as depicted at the top of **Figure 10**) addresses three specific application scenarios: Under weak network conditions, front-end devices with edge computing capabilities perform local pest detection in real-time and autonomously activate laser-based capture mechanisms. Under stable network conditions, low-cost front-end visual sensors transmit images to a backend cloud platform for rapid pest identification, subsequently triggering front-end laser capture devices, thus optimizing deployment costs. Agricultural technicians or unmanned aerial vehicles (UAVs) upload images to the backend platform, enabling precise identification and geolocation-based positioning, supporting flexible mobile monitoring. The backend cloud platform employs parallel computing to achieve millisecond-level processing and feedback, effectively fulfilling diverse scenario requirements and establishing a comprehensive intelligent pest management system encompassing real-time monitoring, rapid identification, precise localization, and targeted pest control.

**Figure 11:**
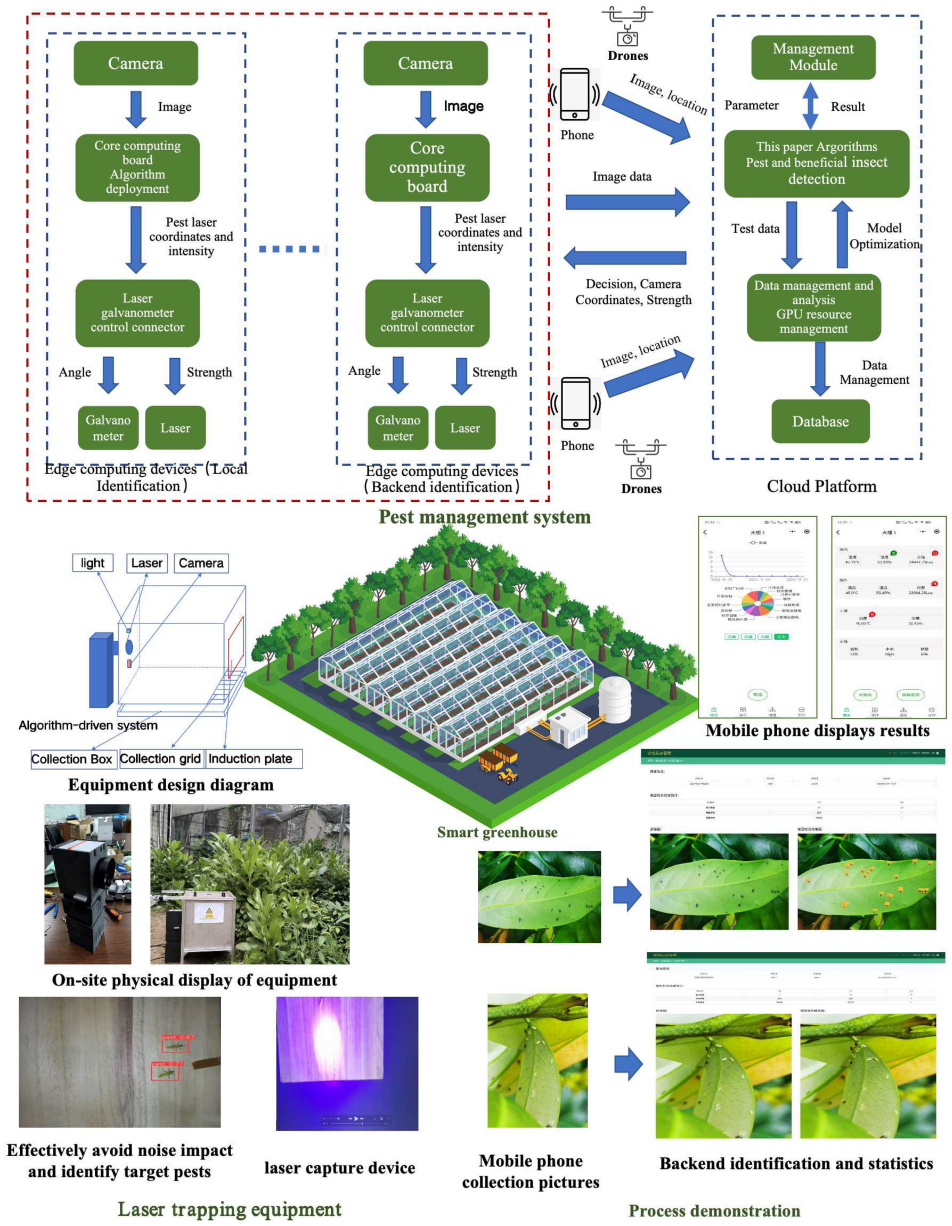
Pest management system architecture, laser trapping equipment and process demonstration.

As shown in the lower-left section of **Figure 11**, the pest induction and laser capture device comprises key modules including a core computing board, laser emitter, galvanometer controller, and visual sensing components. Specific attractants or optical methods accurately lure pests onto designated induction panel areas. Real-time visual data captured by onboard cameras is swiftly processed by a lightweight detection algorithm developed in this research, which can also be deployed in parallel on cloud platforms to handle large volumes of data from multiple devices simultaneously. The coordinate conversion module precisely calculates the physical positions of detected pests, guiding the laser galvanometer to accurately target and activate the laser for pest capture. Captured pests are subsequently collected in designated containers for further identification and analysis. This approach effectively minimizes environmental interference and protects beneficial insects, significantly enhancing the precision and effectiveness of pest monitoring and control.

The backend platform, based on our proposed algorithm, provides a comprehensive management interface, facilitating efficient, real-time collection of pest monitoring data from greenhouses and farms. Data can be flexibly submitted by agricultural technicians via smartphones or automatically uploaded by pest induction and laser capture devices. The backend management system automatically identifies pests, clearly visualizes real-time identification results, and assigns data to corresponding greenhouse or farmland regions according to geographic locations. The detailed system processing workflow is presented in the lower-right section of **Figure 10**.

To further validate the practical efficacy of our proposed few-shot pest insect detection model, we conducted an extensive field evaluation over a three-month period in vegetable greenhouses located in Haikou, Hainan Province. Situated in a tropical region, Hainan faces significant pest challenges. The evaluation specifically targeted eight prevalent pest species in this region: flea beetles, aphids, whiteflies, thrips, diamondback moths, armyworms, fruit flies, and leaf miners. We deployed a detection platform utilizing our proposed algorithm, continuously monitoring pest instances captured through smartphone images provided by agricultural technicians and integrated intelligent trapping devices. Throughout the evaluation period, a total of 563 pest instances were captured across all monitored areas. Among these, the AI model successfully identified 534 instances, yielding an overall accuracy of 94.84%. Notably, aphids and whiteflies demonstrated the highest detection accuracy, each exceeding 96%. In contrast, flea beetles exhibited slightly lower accuracy at 89.7% due to their smaller size and higher mobility.

Our methodology comprehensively addresses the dynamic and complex nature of pest monitoring environments by employing targeted detection strategies that integrate crop types, regional characteristics, and seasonal factors, significantly reducing data collection and labeling costs through few-shot learning techniques. The lightweight model design ensures effective deployment even in agricultural scenarios with limited computational resources or poor network connectivity, exhibiting robust and stable performance in greenhouse monitoring environments.

## 5 Conclusion

This study presents a novel FSOD method for pest insects, addressing challenges related to limited annotation data and multi object sizes. Built upon the Faster R-CNN framework, our approach integrates feature aggregation and SCL to enhance feature representation and improve detection accuracy. Multi-scale feature extraction using a Feature Pyramid Network captures rich semantic information at different scales, improving sensitivity to multi targets. A Feature Aggregation Module (FAM) with attention mechanism fuses features from the support and query sets, enhancing detection ability for small-sample targets. SCL is introduced to improve feature discriminability, while class weights and Focal Loss address class imbalance and hard-to-classify samples. Joint optimization of multiple tasks with an integrated loss function enhances robustness and precision. Experimental results demonstrate significant performance improvements in small and minority class pest detection, offering a valuable solution for agricultural pest management. While the proposed method achieves significant improvements in detection accuracy, the computational cost associated with Faster R-CNN remains a limitation for real-time applications. Future research could focus on optimizing the framework for faster inference or exploring lightweight architectures to enhance scalability for edge deployment.

## Data Availability

The raw data supporting the conclusions of this article will be made available by the authors, without undue reservation.
